# Prediction for Global Peste des Petits Ruminants Outbreaks Based on a Combination of Random Forest Algorithms and Meteorological Data

**DOI:** 10.3389/fvets.2020.570829

**Published:** 2021-01-07

**Authors:** Bing Niu, Ruirui Liang, Guangya Zhou, Qiang Zhang, Qiang Su, Xiaosheng Qu, Qin Chen

**Affiliations:** ^1^School of Life Sciences, Shanghai University, Shanghai, China; ^2^Technical Center for Animal, Plant and Food Inspection and Quarantine of Shanghai Customs, Shanghai, China; ^3^Guangxi Institute for Food and Drug Control, Nanning, China; ^4^National Engineering Laboratory of Southwest Endangered Medicinal Resources Development, Guangxi Botanical Garden of Medicinal Plants, Nanning, China; ^5^Computing Center of Guangxi, Nanning, China

**Keywords:** peste des petits ruminants, Worldclim, random forest algorithm, global online prediction system, outbreaks

## Abstract

Peste des Petits Ruminants (PPR) is an acute and highly contagious transboundary disease caused by the PPR virus (PPRV). The virus infects goats, sheep and some wild relatives of small domestic ruminants, such as antelopes. PPR is listed by the World Organization for Animal Health as an animal disease that must be reported promptly. In this paper, PPR outbreak data combined with WorldClim database meteorological data were used to build a PPR prediction model. Using feature selection methods, eight sets of features were selected: bio3, bio10, bio15, bio18, prec7, prec8, prec12, and alt for modeling. Then different machine learning algorithms were used to build models, among which the random forest (RF) algorithm was found to have the best modeling effect. The ACC value of prediction accuracy for the model on the training set can reach 99.10%, while the ACC on the test sets was 99.10%. Therefore, RF algorithms and eight features were finally selected to build the model in order to build the online prediction system. In addition, we adopt single-factor modeling and correlation analysis of modeling variables to explore the impact of each variable on modeling results. It was found that bio18 (the warmest quarterly precipitation), prec7 (the precipitation in July), and prec8 (the precipitation in August) contributed significantly to the model, and the outbreak of the epidemic may have an important relationship with precipitation. Eventually, we used the final qualitative prediction model to establish a global online prediction system for the PPR epidemic.

## Introduction

PPR also known as pseudorinderpest [as designated by the World Organization for Animal Health (OIE)], is an acute viral infection caused by PPRV. PPRV belongs to the Morbillivirus genus within the Paramyxoviridae family and is closely related to rinderpest virus of the same genus, with only one serotype (1). Affected animals are characterized by symptoms including fever, stomatitis, diarrhea and pneumonia, and these can often be confused with secondary infections, making PPR a serious viral disease that is difficult to characterize, diagnose and treat. PPRV mainly infects small ruminants and the virus does not cause human infection (2). PPRV can be transmitted by direct contact and is highly contagious. Small ruminants are infected by contact with the secretions or feces of infected animals, or by transporting infected animals to uninfected areas (3). Once introduced, the virus can infect up to 90% of herds, and the disease can kill 30 to 70% of infected animals. Since the first outbreak of PPR in Ivory Coast in 1942, it has spread to most parts of Africa, the Middle East, and Asia. To date, more than 70 countries have confirmed the discovery of PPR in their territories, and many are at risk of disease transmission. There are nearly 1.7 billion sheep and goats in these regions, accounting for about 80% of the global total (OIE). The food security and livelihoods of farmers in Africa, the Middle East and Asia have all been affected by the outbreak of PPR, especially affecting the production of small ruminants, which in turn exacerbates some of the world's poorest regional poverty stricken areas and has brought a huge economic burden to agriculture. Currently, in many developing countries, particularly in West Africa and South Asia, PPR is considered to be one of the major animal transboundary diseases that threatens livestock production (4, 5).

A large part of the healthy and sustainable development of animal husbandry is affected by animal diseases. Frequent outbreaks of animal diseases will cause a large number of animal deaths, which will seriously affect income-generating households and businesses that depend on livestock products. Moreover, in order to prevent the further development of the epidemic, relevant government departments will also take corresponding countermeasures, which also brings a huge economic burden (6). Therefore, it is very important to carry out risk assessments on the transmission characteristics and outbreak risks of animal diseases, so that early warning is in advance. The traditional part of veterinary medicine includes: risk assessment and management. The Agreement on Sanitary and Phytosanitary Measures issued by the World Trade Organization in 1994 significantly increased the application of import risk analysis methods (7, 8). A series of mathematical or statistical methods are used in epidemic risk analysis (9). For example, there are risk assessment models based on qualitative or quantitative analysis, spatial prediction models based on geographic information systems (10), propagation dynamics models and prediction models based on machine learning algorithms (11, 12), etc.

Qualitative and quantitative models are two major categories of risk assessment models. Qualitative models often use “high,” “medium,” and “low” to describe the probability of risk occurring. The importance of risk factors is then used to determine the overall degree of risk (13). Assigning values to various elements and potential risks that constitute risks is the key to quantitative models. When all the elements of measuring risk are allocated, the entire evaluation process and results can be quantified (14, 15). In 2015, Woube et al. used the Monte Carlo simulation method to sample the occurrence probability of each risk node based on the risk scenario tree, and assessed the risk of importing bovine pleuropneumonia into the Ethiopian region through the import route (16). The national veterinary department plays a very important role in protecting animal health, providing reliable disease monitor information, and performing scientific and effective assessments (17). However, veterinary services in many countries are now unable to perform their disease monitoring, prevention, and control duties. Because of the lack of veterinary services, and animal disease surveillance methods are still very traditional (18). At the same time, due to the differences in monitoring methods of different countries, it usually leads to delays in reporting, which will increase the spread of the epidemic and cause irreparable losses.

With the rapid development of machine learning technology, more and more people have started to pay attention to regression and classification problems that can help them find the rules governing massive data in order to achieve the prediction effect. Data prediction has become an important part of daily life. The technology has been widely used in weather forecasting, medical diagnosis and financial forecasting. Machine learning is a multidisciplinary major covering probability theory, statistics and computer algorithms. Ability to learn from data through continuous optimization algorithms for analysis and prediction (11, 19, 20). Machine learning is currently widely used in different research fields, such as economics, biomedicine, engineering technology, etc. At the same time, machine learning can be used to solve classification, regression, clustering and other problems according to the characteristics of the algorithm (21). In the field of veterinary epidemiology, the epidemic prediction model of machine learning algorithms is gradually being widely used. The model can make an early prediction of the occurrence of the epidemic, because once it breaks out, the destructiveness is incalculable (22). Williams et al. used random forest (RF) algorithms to predict suitable habitats for six rare plant species in Creek Terrane, Northern California (23). In 2013, the enhanced regression tree algorithm was used by Samir Bhatt et al. to establish a dengue risk statistical model to predict dengue epidemics worldwide (24).

In addition, climate and environmental factors (temperature, precipitation, etc.) have an impact on the spread of the virus, and climate and environmental factors may be the main cause of the occurrence and recurrence of infectious diseases. The analysis of the relationship between environmental and climatic conditions and diseases plays an important role in the monitoring and control of animal epidemics. The WorldClim database is based on monthly average climate data for weather stations from a large number of global, regional, national, and local sources (25). The WorldClim data set is widely used in the simulation of global climate change and disease analysis. In 2019, Zheng et al. conducted seasonal simulation of the environmental suitability distribution of Aedes albopictus in China based on Worldclim data (26). Chalghaf et al. used temperature and precipitation data from the worlsclim database to predict the distribution of Leishmania vetors in the Mediterranean Basin using a comprehensive niche model method (27). Mollalo et al. collected remote sensing data and meteorological data related to environmental factors under the framework of geographic information System (GIS). They used different machine learning algorithms to build a qualitative prediction model for leishmaniasis, and the results showed that support vector machine (SVM) algorithm had the best modeling effect (28).

Inspired by the successful application of these prediction models in disease surveillance, we want to apply them to the prediction of PPR. In this paper, PPR outbreak data and WorldClim database meteorological data were used to build a PPR prediction model. After feature selection methods were used, eight sets of features were selected: bio3, bio10, bio15, bio18, prec7, prec8, prec12, and Alt for modeling. Then different machine learning algorithms were used to build models, among which the RF algorithm was found to have the best modeling effect. Therefore, the RF algorithms of eight variables was finally selected to build the model. In addition, in order to determine the impact of each variable on the modeling results, we performed single-factor modeling and correlation analysis on the variables involved during the modeling. Eventually, we used the final qualitative prediction model to establish a global online prediction system for the PPR epidemic. On this basis, we hope that this research can play a role in monitoring the outbreak of PPR in the future. Thereby reducing the occurrence of epidemics and economic losses.

## Materials and Methods

### Source and Pretreatment of Data

*Outbreak data*: Information on the PPR outbreak was obtained from the global Animal Disease Information System (Empres-I) of the Food and Agriculture Organization of the United Nations (FAO), including the specific time of outbreak, the longitude and latitude of the outbreak point. The time range of outbreak data used to construct the global PPR prediction model was January 1, 2008 to December 31, 2018.

*Climate data***:** 19 global climate-related variables (bio1–19) and precipitation (prec1–12 month) and altitude data were collected from the WorldClim database of WorldClim version 2 (http://www.worldclim.org).

### Methods

#### Naïve Bayes

The Naïve Bayes (NB) belongs to the supervised learning generative model and is a simple probability classifier based on Bayes' theorem (29). The algorithm consists of two types of probabilities: the probability that the data belongs to each class and conditional probability. Once these two probabilities have been calculated, the Bayesian probability model can be used to predict the new data. It has the advantages of simple implementation, no iteration, and high learning efficiency (30). Therefore, in practical applications, NB classifier is widely used to solve classification problems.

#### Random Forest

Random forest (RF) is an algorithm developed and inferred by Breiman et al. (31). In machine learning, a random forest is a classifier that contains multiple decision trees. It can handle a large number of input variables and can produce a highly accurate classifier (32). A random forest is an improvement on this approach, which creates a decision tree so that the optimal segmentation point is not selected, but suboptimal segmentation is performed by introducing randomness.

#### AdaBoost

The AdaBoost (Adaptive Boosting) is an integrated learning algorithm proposed by Freund and Schapire to solve dichotomy. It can adaptive to adjust the weight distribution of samples, and set the weight of the wrong samples high and the weight of the right samples low (33). In summary, After AdaBoost creates the first tree, it uses the training performance of this instance to measure how much weight should be given to each training instance in the next tree. The weight of training data that is hard to predict will increase, while the weight of instances that are easy to predict will decrease. The models are created one by one, and each model updates the weight of the training instance, which affects the learning of the next tree in the sequence. The research and application of AdaBoost algorithm are mostly concentrated on classification problems, and have achieved great success in solving application problems in different industries.

#### Support Vector Machine

Support vector machine (SVM) is a generalized linear classifier proposed by Vapnik et al. (34). In simple terms, the basic steps of SVM are as follows: first map the input points to the high-dimensional feature space and find the separated hyperplane to maximize the margin between the two classes in this space. The marginal distance between the decision hyperplane and the instances that are closest to boundary is maximized (35). SVM is widely used in classification algorithms, and the classification effect is very good. For example, it is used in pattern recognition problems such as portrait recognition and text classification. It has unique advantages in small samples and high-dimensional data analysis (36).

#### Artificial Neural Networks

Artificial neural network (ANN) is a powerful machine learning model. It is a complex network structure formed by simulating a large number of processing units connected to each other based on a mathematical model (37). The input layer, the hidden layer and the output layer are three parts of the neural network. The more layers of hidden layer, the more nodes of hidden layer, under the nonlinear activation function, the neural network can learn deeper features. The artificial neural network has self-learning, adaptability and has strong fault tolerance. It is a powerful tool for handling non-linear systems.

#### C4.5

C4.5 is a machine learning algorithm for classification decision based on ID3, which improves the objective function of split attributes (38). C4.5 algorithm uses information gain rate instead of information gain as the attribute selection criteria of decision tree. In addition, it has the advantages of processing discrete and continuous attribute types, pruning after constructing decision trees, and processing training data with missing attribute values. Because of its simplicity and high performance, C4.5 is a very important algorithm to implement accurate classification models.

#### Feature Selection

Features are the performance of some prominent properties and the key to distinguishing things. Therefore, when we want to classify or identify things, we actually judge by the performance of the features. When the data is redundant and complex, feature selection can be selected from the original attribute data set, identifying and deleting as much irreparable and redundant information as possible. It simplifies the size and complexity of the data set and can increase the effect of classification. So as to better solve the problem.

### Calculation

#### Analysis Tools

Weka version 3.6 can be used for machine learning algorithm modeling and analysis (https://www.cs.waikato.ac.nz/ml/weka/), and this runs under the Windows 7 64-bit operating system. In this study, the parameter settings of all the machine learning algorithms used are summarized in Supplementary Material I.

#### Construction of an Online Prediction System

J2EE and MVC frameworks were used to construct a qualitative prediction model of PPR. The three-layer architecture of view layer, control layer and model layer realizes the separation of data display and data processing. The specific implementation of the system is as follows:

*a. View layer*: The Worldclim data input by the user is first transmitted to the control layer in the form of a data stream, and then the prediction results of the control layer are output to realize the visualization results.

*b. Control layer*: Call the function to transfer the data instance to the model layer and output the calculation result of the model layer to the view layer.

*c. Model layer:* The model layer is an important core module, and the model calculation is performed by reading the data instances generated by the control layer.

## Results

### Analysis of the Global PPR Outbreak

PPR is an infectious transboundary disease that has a severe economic impact on people who depend on income from livestock products. The small ruminant epidemic was first reported in Ivory Coast in 1942, and then spread rapidly. The epidemic has occurred in 70 countries around the world, covering Asia, Africa, the Near East, and the Middle East.

**Figure 1:**
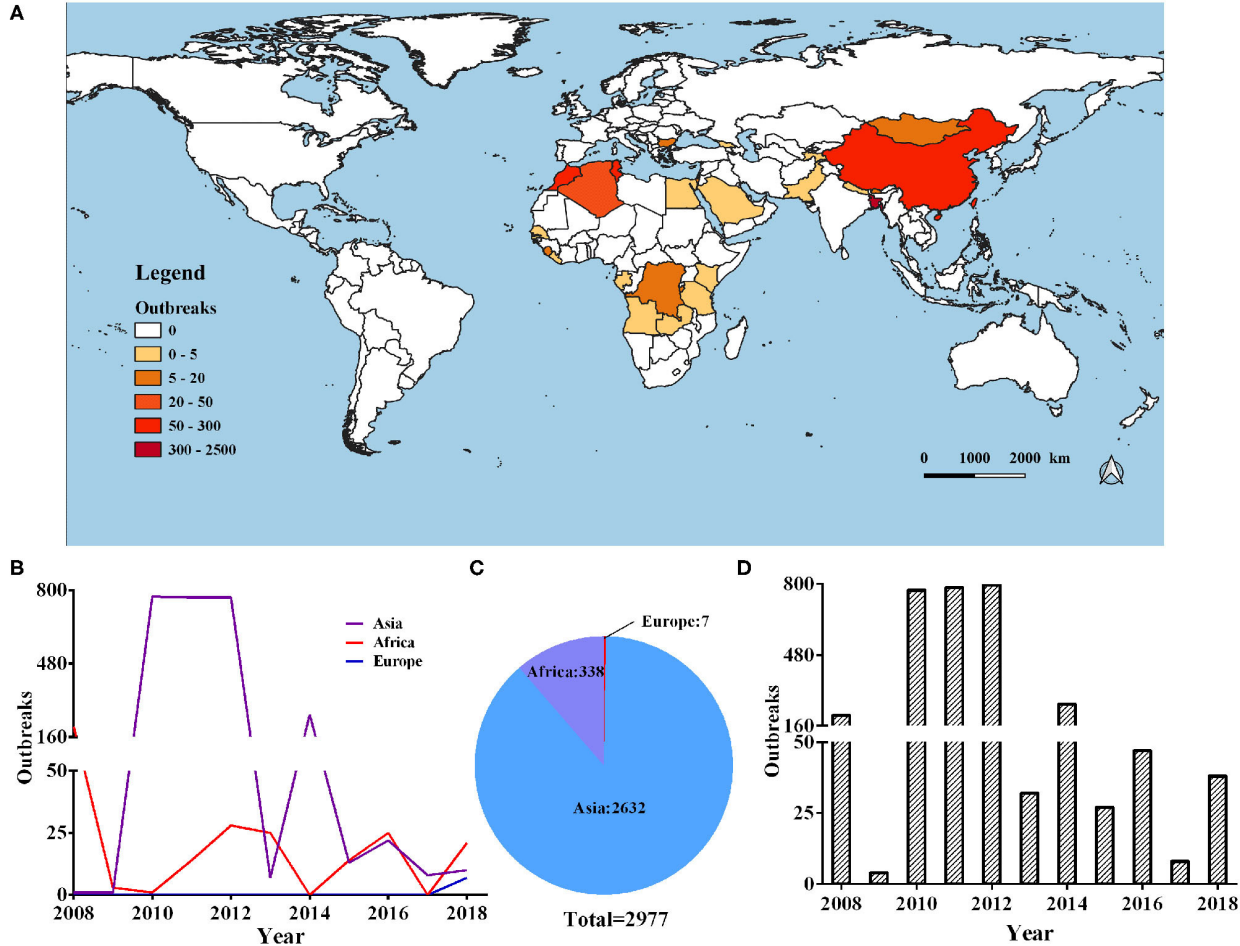
Global distribution of PPR during 2008–2018. **(A)** Heat map of the global PPR epidemic outbreaks between 2008 and 2018 inclusive. **(B)** Number of PPR outbreaks in Europe, Asia, and Africa during 2008–2018. **(C)** Number of PPR outbreaks per year from 2008 to 2018.

In our current research, we collected data from 2,977 cases of PPR outbreaks in the world from 2008 to 2018 from Empres-I system (detailed outbreak data are not available in some countries). Based on this data analysis, we found that the outbreaks were concentrated on three continents in Europe, Asia, and Africa. The global PPR epidemic is still erupting frequently and has an upward trend (as shown in Figure 1). The Europen Food Safety Authority (EFSA) considers PPR to be one of the key animal diseases that should be controlled in Africa, the Middle East and South Asia, and is considered essential for poverty alleviation in these regions.

### Identification of Outbreak Sites

**Figure 2:**
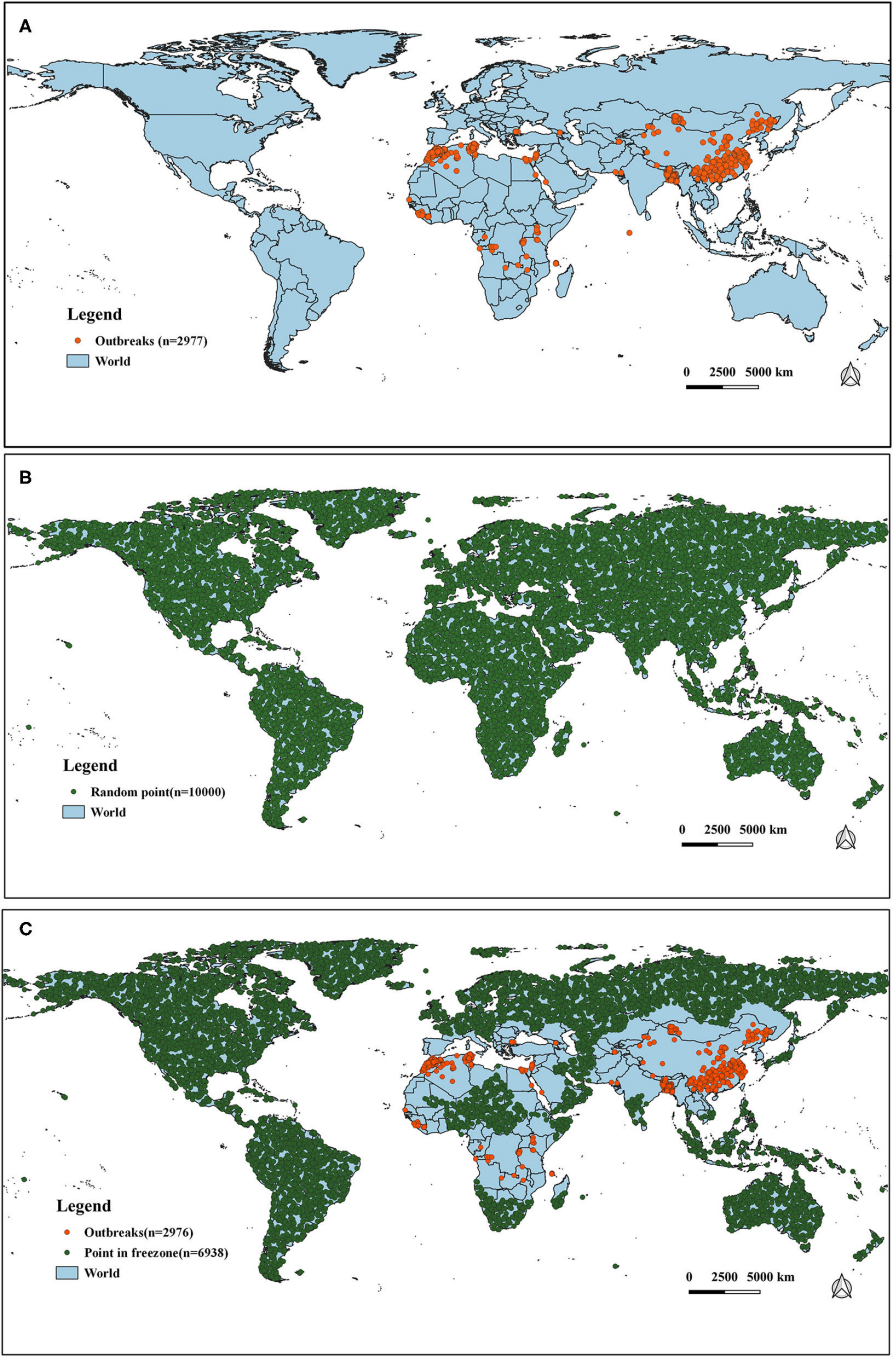
The distribution of outbreak and non-outbreak points in the study region. **(A)** Global outbreak points of PPR (*n* = 2,977). **(B)** Selection of random points (*n* = 10,000). **(C)** Distribution of outbreak points (*n* = 2,976) and non-outbreak points (*n* = 6938).

It has long been observed that diseases are closely related to seasons and climate. Changes in weather can often cause changes in certain diseases, especially those caused by certain viruses. The survival and spread of viruses are often related to climatic variables such as temperature. In order to study the impact of temperature and precipitation on the distribution of PPR epidemics, we obtained 32 global climate-related variables: bioclimatic variables (bio1–bio19), precipitation from January to December (prec1–12) and altitude data (alt). WorldClim version 2 has the average monthly climate data for the minimum, mean, and maximum temperatures and for precipitation observed from 1970–2000. The specific meanings of these variables are shown in Table 1 [the spatial resolution of the variables is 30 s (~1 km^2^)]. The outbreak data used in this study was collected from the EMPRS-i disease information system, and these defined 2,976 points (excluding a coordinate point in the Maldives) as the outbreak points (Figure 2). Then we used the R language code to randomly generate 10,000 random points in the global scope, removed the above outbreak points and the data points located in the Antarctic continent, and set the remaining 6,938 points as non-outbreaks points. (The original data of this study are summarized in Supplementary Material II).

**Table 1:** The variables and description of data in the WorldClim database.

**Variable**	**Description**	**Variable**	**Description**
bio1	Annual mean temperature	bio12	Annual precipitation
bio2	Mean diurnal range [mean of monthly (max–min temp)]	bio13	Precipitation of wettest month
bio3	Isothermality (bio2/bio7) (*100)	bio14	Precipitation of driest month
bio4	Temperature seasonality (standard deviation *100)	bio15	Precipitation seasonality (coefficient of variation)
bio5	Max temperature of warmest month	bio16	Precipitation of wettest quarter
bio6	Min temperature of coldest month	bio17	Precipitation of driest quarter
bio7	Temperature annual range (bio5–bio6)	bio18	Precipitation of warmest quarter
bio8	Mean temperature of wettest quarter	bio19	Precipitation of coldest quarter
bio9	Mean temperature of driest quarter	Prec1–12	Precipitation from January to December
bio10	Mean temperature of warmest quarter	alt	altitude
bio11	Mean temperature of coldest quarter		

Finally, climate variables of the above outbreaks and non-outbreaks were extracted from the WorldClim version 2 database to construct the data set, which was divided into a training set (*n* = 5948, in which 1,783 outbreaks and 4,165 non-outbreaks occurred, respectively) and a test set (*n* = 3,966, in which 1,193 and 2,773 are outbreaks and non-outbreaks occurred, respectively) for model construction.

### Feature Selection

When we classify or recognize things, we actually judge them by their features. For example, for a data set with n variables, there may be 2^n^ combinations of subsets. Due to the huge amount of computation, we cannot try all methods, so feature selection becomes very important. In line way many feature selection methods, this study adopted the different properties discriminator in combination with different search methods to search for the optimal feature subset, explored the possible combinations of common ones and selected 1–32 different subsets of combination. The results are shown in Table 2, and by comparing the variable selection results, we found that screening of variables for 8, 11, and 15 was the most reasonable avenue to pursue and selection of the types of variables stayed consistent. Therefore, we finally chose to filter the number of variables as 8, 11, and 15 variables to form the dataset for the modeling process.

**Table 2:** Feature selection for screening of different variables.

**Attribute evaluator**	**Search method**	**No**.	**Variable**
CfsSubsetEval	BestFirst	8	3,10,15,18,26,27,31,32
	GeneticSearch	11	3,6,10,15,18,24,26,27,28,31,32
	GreedyStepwise	8	3,10,15,18,26,27,31,32
	LinearForwardSelection	8	3,10,15,18,26,27,31,32
	RankSearch	15	1,3,4,9,10,11,13,15,16,18,25,26,27,28,31
	ScatterSearchV1	8	3,10,15,18,26,27,31,32
	SubsetSizeForwardSelection	8	3,10,15,18,26,27,31,32
ChiSquaredAttributeEval	Ranker	32	all
ClassifierSubsetEval	GeneticSearch	1	25
	RankSearch	1	27
ConsistencySubsetEval	BestFirst	8	5,7,9,15,19,22,23,27
	GeneticSearch	10	3,6,10,14,18,20,22,28,30,32
	GreedyStepwise	8	5,7,9,15,19,22,23,27
	LinearForwardSelection	8	5,7,9,15,19,22,23,27
	RankSearch	20	1,3,4,5,6,8,9,10,11,12,13,15,16,18,24,25,26,27,28,31
	SubsetSizeForwardSelection	8	5,7,9,15,19,22,23,27
FilteredAttributeEval	Ranker	32	all
FilteredSubsetEval	BestFirst	8	3,10,15,18,26,27,31,32
	GeneticSearch	11	3,6,10,15,18,24,26,27,28,31,32
	GreedyStepwise	8	3,10,15,18,26,27,31,32
	LinearForwardSelection	8	3,10,15,18,26,27,31,32
	RankSearch	15	1,3,4,9,10,11,13,15,16,18,25,26,27,28,31
	ScatterSearchV1	8	3,10,15,18,26,27,31,32
	SubsetSizeForwardSelection	8	3,10,15,18,26,27,31,32
GainRatioAttributeEval	Ranker	32	all
InfoGainAttributeEval	Ranker	32	all
LatentSemanticAnalysis	Ranker	1	1
OneRAttributeEval	Ranker	32	all
PrincipalComponents	Ranker	8	1,2,3,4,5,6,7,8
ReliefFAttributeEval	Ranker	32	all
SVMAttributeEval	Ranker	32	all
SymmetricalUncertAttributeEval	Ranker	32	all
WrapperSubsetEval	GeneticSearch	1	25
	RankSearch	1	27

### Prediction Model Construction

After the feature selection in the previous step, the 8, 11, and 15 variables were selected to form the dataset for the subsequent modeling. During the model construction process, we selected 10 common machine learning algorithms (such as SVM, KNN, RF, etc.) for modeling, and evaluated the model in terms of sensitivity (SN), specificity (SP), prediction accuracy (ACC) and Matthews' coefficient (MCC). As shown in Table 3. When eight variables were involved in the modeling, the prediction accuracy using 10 algorithms was within range of 93.1–99.6% on the training set and 92.8–99.5 on the test set. With these methods, the KNN (99.6, 99.5%) and RF (99.1, 99.1) algorithms had the highest ACCS on both the training and test sets. When 11 variables were involved in the modeling of 10 algorithms, the ACC value of the modeling prediction accuracy on the training set was 92.3–99.4%, and for the test set was 92.0–99.7%, with KNN (99.4, 99.7%) and RF (99.2, 99.3%) algorithms having the highest prediction accuracies on both the training and test sets, respectively. In addition, when 15 variables were used to model 10 algorithms, the ACC value on the training set was 91.9–99.4%, and on the test set was 91.7–99.7%, with the KNN (99.4, 99.7%) and RF (99.2,99.2%) algorithms again having the highest prediction accuracies. Therefore, when the number of variables was eight and then modeling effect achieved was the best. Finally, the eight variables that were selected for modeling were, bio3, bio10, bio15, bio18, prec7, prec8, prec12, and Alt.

**Table 3:** Weak classification filter results for classification modeling of the variables.

**Number**	**Algorithm**	**Training set**				**Test set**			
		**SN**	**SP**	**ACC**	**MCC**	**SN**	**SP**	**ACC**	**MCC**
8	BayesNet	80.99	98.63	93.34	0.84	82.06	98.67	93.67	0.85
	NaiveBayes	80.76	98.49	93.17	0.84	81.22	97.84	92.84	0.83
	ANN	96.63	99.09	98.35	0.96	96.65	99.86	98.89	0.97
	SVM	86.37	99.54	95.6	0.9	85.92	99.21	95.21	0.89
	KNN	99.89	99.52	99.63	0.99	99.25	99.64	99.52	0.99
	Adaboost	83.01	98.25	93.68	0.85	84.91	97.26	93.55	0.84
	Bagging	96.3	99.54	98.57	0.97	95.31	99.5	98.23	0.96
	BFTree	96.3	99.04	98.22	0.96	96.9	98.85	98.26	0.96
	C4.5	96.63	99.26	98.47	0.96	97.07	99.42	98.71	0.97
	RF	97.53	99.81	99.13	0.98	97.74	99.82	99.19	0.98
11	BayesNet	79.02	98.8	92.87	0.83	80.55	98.74	93.27	0.84
	NaiveBayes	79.75	97.72	92.33	0.81	80.64	96.93	92.03	0.81
	ANN	97.25	99.5	98.82	0.97	97.32	99.6	98.92	0.97
	SVM	85.59	99.57	95.38	0.89	85.58	99.35	95.21	0.89
	KNN	99.05	99.64	99.46	0.99	99.58	99.82	99.75	0.99
	Adaboost	83.85	97.09	93.12	0.83	84.91	97.26	93.55	0.84
	Bagging	96.47	99.59	98.66	0.97	95.31	99.57	98.29	0.96
	BFTree	96.63	99.09	98.35	0.96	95.31	99.31	98.11	0.95
	C4.5	97.25	99.21	98.62	0.97	97.23	99.39	98.74	0.97
	RF	97.87	99.88	99.28	0.98	97.9	99.93	99.32	0.98
15	BayesNet	79.98	98.73	93.11	0.83	81.31	98.23	93.14	0.83
	NaiveBayes	80.54	96.81	91.93	0.80	81.89	95.96	91.73	0.80
	ANN	97.36	99.54	98.89	0.97	96.65	99.5	98.64	0.97
	SVM	84.3	99.62	95.02	0.88	84.58	99.53	95.03	0.88
	KNN	98.93	99.66	99.45	0.99	99.5	99.78	99.7	0.99
	Adaboost	83.51	96.97	93	0.83	84.91	97.26	93.55	0.84
	Bagging	97.03	99.57	98.81	0.97	95.73	99.28	98.21	0.96
	BFTree	96.41	99.26	98.4	0.96	96.14	99.35	98.39	0.96
	C4.5	96.92	99.3	98.59	0.97	97.49	99.39	98.81	0.97
	RF	97.81	99.86	99.24	0.98	97.65	99.93	99.24	0.98

### Model Prediction

To verify the data obtained before we set up the accuracy of the model, we chose a global outbreak of small ruminants which occur frequently in three countries: China, Bangladesh, and Morocco, and the outbreak data of 2008–2018 from the FAO was downloaded as positive sample. This was randomly generated in the country without breaking point as negative samples was set up as three separate independent test sets. The variables filtered were selected, and the data preprocessing was carried out in order to establish the model for verification.

The prediction results obtained are shown in Table 4. The accuracy of the model was 60.2 to 73.3% for the Chinese verification test set, 55.4 to 100% for the Moroccan verification test set, and the lowest ACC of 47.8 was obtained for Bangladesh. The ACCs of China and Morocco were higher than that of Bangladesh, where SN was high but SP was low. This may be explained that Bangladesh is a small country (14.757^2^ km) and there was a nationwide outbreak of PPR. Positive and negative samples cannot be separated, while the climates of negative and positive samples (the outbreak point) were similar. In the previous studies, the KNN and RF have the best modeling effect on both the training and test sets. Combined with the validation results from China and Morocco, we found that the RF (73.3, 99.6%) algorithm was more accurate than the KNN (60.7, 88.4%) in the ACC prediction on the test set in the model validation. Comparatively speaking, KNN algorithm has high time complexity and space complexity. The unbalanced sample distribution will lead to misclassification. In the training process, RF algorithm can detect the interaction between features, so as to balance the errors. In the actual classification prediction of this study, RF algorithm has a higher prediction accuracy for different independent test sets, and KNN algorithm is vulnerable to data influence. So in the end, we chose the data set of eight variables (bio3, bio10, bio15, bio18, prec7, prec8, prec12, and Alt) and used the RF algorithm to build the online prediction model of small PPR outbreaks around the world.

**Table 4:** Validation results for China, Bangladesh, and Morocco.

**Algorithm**	**China (%)**			**Bangladesh (%)**			**Morocco (%)**		
	**SN**	**SP**	**ACC**	**SN**	**SP**	**ACC**	**SN**	**SP**	**ACC**
BayesNet	33.5	96.2	66.7	100	0	47.8	33.0	93.4	55.4
NaiveBaye	28.9	96.2	64.5	100	0	47.8	0	100	57.8
ANN	98.9	44.8	70.3	100	0	47.8	100	100	100
SVM	88.4	92.8	90.7	100	0	47.8	0	100	57.8
KNN	100	25.7	60.7	100	0	47.8	99.0	80.6	88.4
Adaboost	70.1	95.9	83.7	100	0.1	47.8	0	100	57.8
Bagging	95.8	77.4	86.1	100	0	47.8	95.2	100	98.0
BFTree	95.4	45.8	69.2	100	0.1	47.8	98.6	88.2	92.6
C4.5	94.7	29.5	60.2	100	1.4	48.5	99.0	99.0	99.0
RF	98.9	50.5	73.3	100	0	47.8	99.0	100	99.6

### Comparison and Analysis of Variables Before and After Screening

**Figure 3:**
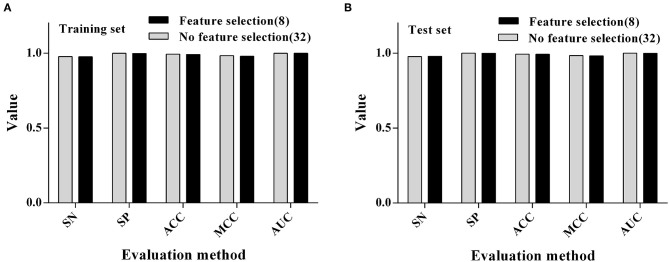
Comparison of modeling results using RF algorithms before and after feature selection. **(A)** Training set. **(B)** Test set.

After the previous variable screening, the RF algorithm was finally selected for the modeling process, and it was found that the ACC value of the model was more than 90%. We also want to further compare the difference between the RF modeling results with no variable screening (i.e., all the variables which were involved in the modeling) and the RF modeling results of previous variable screenings. The results (as shown in Figure 3) showed that the ACC of the model did not decrease when the number of variables was reduced by 75% (from 32 to 8) after variable selection, indicating that the feature selection could process the data to some extent and remove some redundant variables, which could play a corrective role and improve the longitude of the model.

**Figure 4:**
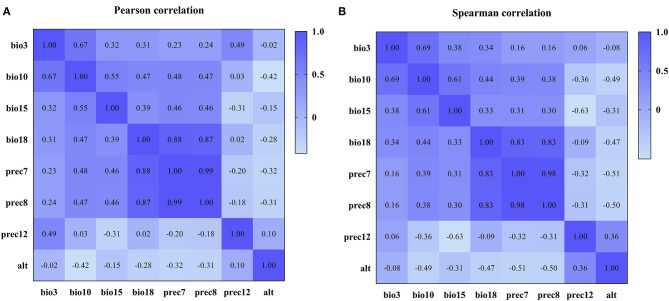
Correlation coefficients among variables after variable screening. **(A)** Pearson correlation coefficient. **(B)** Spearman correlation coefficient.

**Figure 5:**
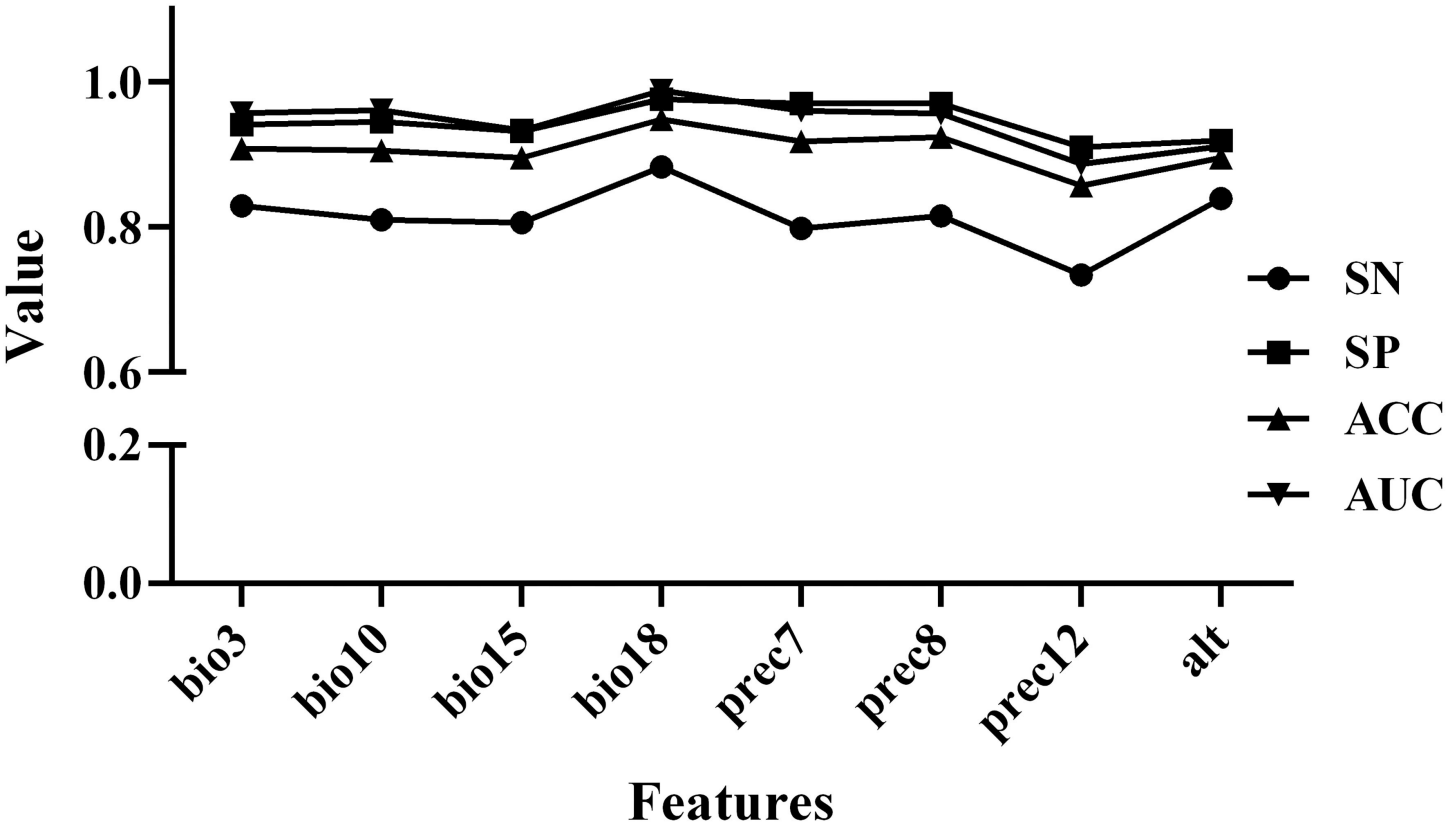
Results of single-factor modeling using RF algorithms.

Based on the construction of the prediction model, we conducted Pearson and Spearman correlation analyses on the eight variables screened for in order to determine the correlation of each variable to the PPR outbreak, and found that the variables selected for had a strong correlation (as shown in Figure 4). In addition, in order to determine the impact of each variable on the modeling results, we conducted a single factor modeling analysis. The results are shown in Figure 5. An estimate of eight variables modeled using the RF method were obtained from the SN, SP, ACC and the area under the ROC curve (AUC). It was found that bio18 (the warmest quarterly precipitation), prec7 (the precipitation in July), and prec8 (the precipitation in August) contributed significantly to the model, and the outbreak of the epidemic may have an important relationship with precipitation. The AUC values for bio2, bio17, prec2, and prec11 were lower, suggesting that these variables had less impact on the outbreak.

### Online Prediction System for PPR

**Figure 6:**
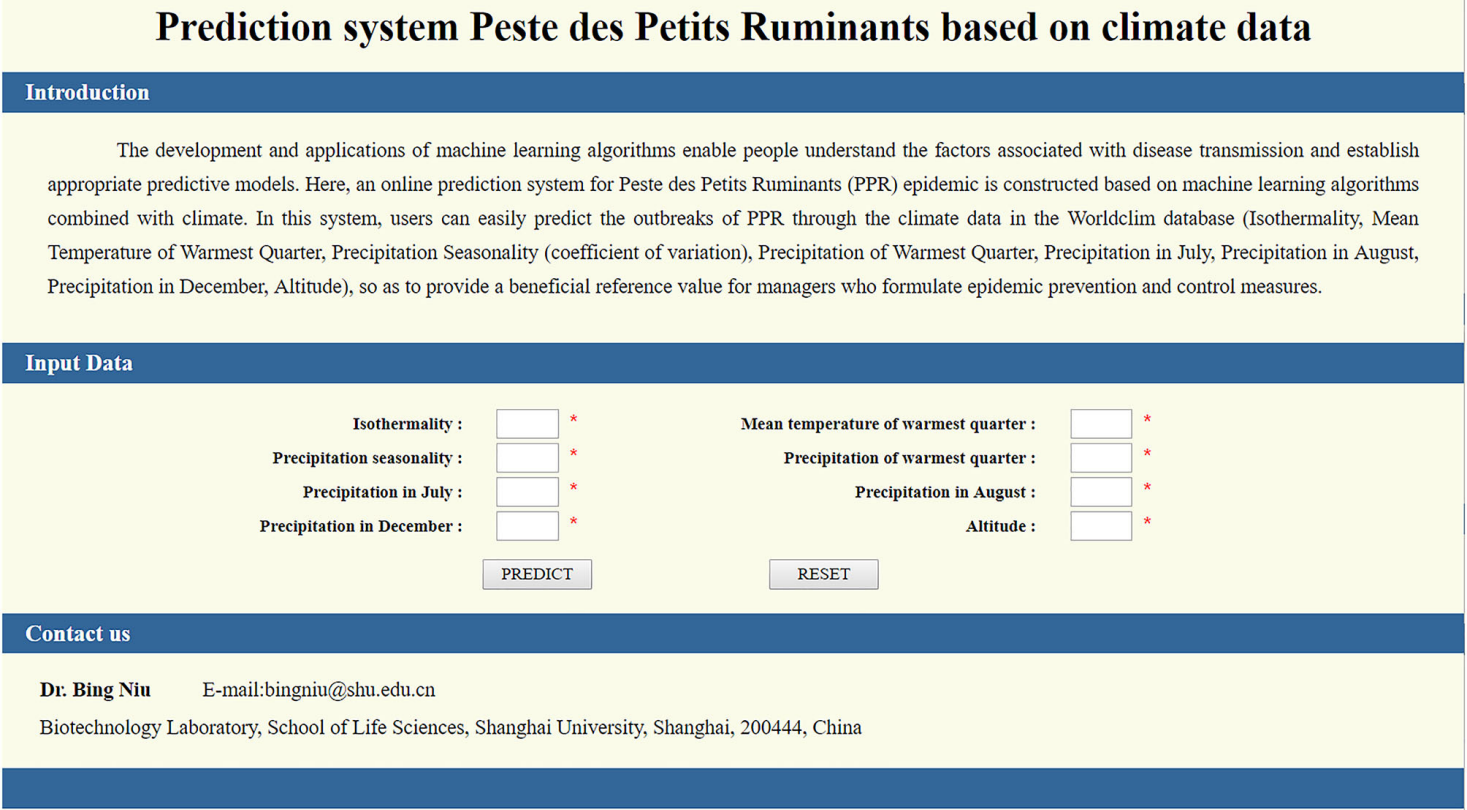
The interface of the Peste des petits ruminants predicting web server.

In view of the superior predictive ability of this epidemic qualitative prediction model, we have further constructed an online prediction system for the use of experimental technicians. Users can access the system through the link http://www.biotechshu.com:8080/PPR/index.jsp (as shown in Figure 6). The operation steps are as follows: Step 1: Enter the initial interface of the prediction system through the website link; Step 2: Enter the value of eight meteorological datasets (bio3, bio10, bio15, bio18, prec7, prec8, prec12, and Alt) in a someone region, and click the “PREDICT” button; Step 3: The system calls the model calculation in the background, and outputs the model result as safety or outbreak.

To sum up, when the user enters the main interface of the system and enters eight relevant meteorological data values, the system will call the model in the background and output the prediction results through the model calculation. The machine learning algorithm is a good analysis tool in understanding the factors related to the spread of disease, and can establish an appropriate prediction model. We hope that this system can play a role in monitoring the outbreak of PPR in the future. Thereby reducing the occurrence of epidemics and economic losses.

## Discussion

Infectious diseases are a type of diseases that are transmitted between humans or animals. For most infectious diseases, the pathogen, the vector (or host) and the transmission environment are three essential elements. The survival, reproduction, distribution and transmission of pathogens, vectors, and hosts require suitable climatic and weather conditions (39). Some studies have shown that changes in the climate and environment may affect the spread of diseases, such as temperature, wind, precipitation, and sunshine (40, 41). For example, climate change will damage the immunity and disease susceptibility of humans or animals, thereby affecting the spread of diseases. In general, climatic conditions limit the geographic distribution of infectious diseases, and weather affects the time and intensity of disease outbreaks (42). The current global infectious disease situation is still severe, and infectious disease surveillance has played an irreplaceable role in controlling the outbreak of infectious diseases (43–45). The establishment of surveillance and prediction models for infectious diseases will still be an effective way to prevent and control infectious diseases. An outbreak of PPR poses a serious threat to small ruminant farming, especially for families who rely on rearing sheep for their income. Based on this, we consider establishing a prediction model of PPR for outbreak surveillance of this disease. Some researches have shown that understanding disease-related factors is a critical step in control and eradication strategies. The machine learning algorithm is one of the analysis methods, and an appropriate algorithm can be used to establish an appropriate prediction model. For example, the RF algorithm was used by Machado et al. to analyzed the importance of factors related to the occurrence of bovine verbal diarrhea vs. (BVDV) (46). Based on the species distribution model, Sehgal et al. further applied machine learning algorithms to determine the relationship between parasite prevalence and environmental predictors (47).

Inspired by the successful application of these prediction models in disease surveillance, we want to apply them to the prediction of PPR. In line with many feature selection methods, this study adopts the different properties discriminator in combination with different search methods to search the optimal feature subset, and then explores the possible combinations of common ones and subsequently selects 1–32 different subsets of combinations. By comparing the variable selection results, it was found that screening of variables for 8, 11, and 15 was a reasonable way to proceed and the selection of the types of the variables were consistent. Therefore, we finally chose to filter the number of variables as 8, 11, and 15 variables to form the dataset for modeling. When eight variables were involved in the modeling, the prediction accuracy of 10 algorithms was within the ACC range of 93.1–99.6% on the training set and 92.8–99.5% on the test set. In conjunction with this, the KNN (99.6, 99.5%) and RF (99.1, 99.1%) algorithms had the highest prediction accuracy on both the training set and test sets. In addition, we conducted Pearson and Spearman correlation analyses on the eight variables screened for, in order that explore the correlation of each variable to the PPR outbreak, and found that the variables selected for, had a strong correlation. Finally, the eight variables scenario was selected for modeling. By comparing the modeling results before and after variable screening, it can be found that feature selection method accelerates the calculation speed of the model and improve the ACC of the model.

Raising the speed of the model calculation is done on the basis of different algorithms with different modeling precisions. Among them, choosing variables screen after eight subsets using the RF algorithm to construct model, the model on the training set achieved the highest ACC of 99.10%. It was found that the ACC values of the KNN and RF algorithms were relatively close, but the prediction accuracy of the RF algorithm in the model validation for China and Morocco was better than that of the KNN algorithm (60.7, 88.4%) and the ACC value (73.3, 99.6%). This may depend on its ability to process very high-dimensional data. For example, when there are a large number of unknown features in the data set, it can well-detect the interaction between features and which features are more important in the classification process. Even when there is a lot of noise in the data set, good prediction performance can be achieved.

Machine learning is a branch of artificial intelligence (AI), and its statistical learning ability provides important help for the diagnosis, animal population monitoring and analysis of in veterinary epidemiology (48, 49). With limited laboratory testing capabilities, AI approaches can rapidly monitor and analyze animal epidemic diseases such as avian influenza, African swine fever (ASF) (49). Walsh et al. successfully applied gradient boosted trees combined with features of avian influenza (bird type, age, etc.) to predict the probability of isolating avian influenza viruses (AIV) (50). Fernandez-Carrion et al. applied AI innovation technologies including deep learning and computer vision to detect lethargy of ASF among wild boars under experimental conditions. These investigation suggest the potential application of AI algorithms in veterinary epidemiology (51). In this study, we used 10 different machine learning algorithms for modeling outbreaks of ASF, and our results showed that the application of machine learning modeling and available dataset (outbreaks data and meteorological data) in the public domain are promising. We think this approach could also be used to guide for understanding other infectious diseases, such as foot and mouth disease and African swine fever research, and so on. For most animal epidemic diseases, climate change is only one of many factors affecting the spread of disease. The occurrence of animal epidemic diseases is the result of the mutual influence of various internal and external factors. In addition, the density of livestock population, the age of livestock, and the difference of geographical location are also external factors that we cannot ignore. Peste des Petits Ruminants is a serious disease of small ruminants, which is exacerbated by animal migration, trade, and increased breeding density (52). In livestock industries, the spatial distribution data of the animal population and farm is very important for the risk management of relevant government departments (53). Hollings et al. assessed three machine learning [boosted regression trees (BRT), random forests (RF), and K-nearest neighbor (K-NN)] species distribution models (SDM) for their capacity to estimate national-level farm animal population numbers within property boundaries. Cecilia et al's. evaluation model based on machine learning algorithms found that host data (farm density) with vector abundance predictions was sufficient to identify areas at greater risk of becoming endemic (54).

In our following research, the population density and distribution of susceptible animals, the movement and migration of animals, and other features should also be taken into account in future researches. What's more, if the data set is sufficient, we can increase the comparison between algorithms and use new algorithms such as deep learning. In addition, the potential errors and systematic errors of the data set should also be considered. We believe that these can increase the accuracy of classification prediction. In addition, in order to determine the impact of each variable on the modeling results, we selected the optimal RF algorithm for single-factor modeling and determined the prediction and contribution analysis of each variable's characteristics to the PPR outbreak. It was found that bio18 (the warmest quarterly precipitation), prec7 (the precipitation in July), and prec8 (the precipitation in August) contributed significantly to the model, and the outbreak of the epidemic may have an important relationship with precipitation. The AUC values of bio2, bio17, prec2, and prec11 were lower, suggesting that these variables had less impact on the PPR outbreak. These three variables are more important predictors of PPRV outbreak than other variables, and there was a relationship between meteorological factors and PPR outbreak, which was also consistent with the research results of Ma Jun et al. (55, 56). PPRV is a kind of enveloped virus, which is more stable in a dry environment (57). So, we have further constructed an online prediction system for the use of experimental technicians. The system plays a role in risk monitoring. It can be used to monitor risk trends in different regions and make an overall forecast of PPR outbreaks based on climate variables. Based on the nature of machine learning algorithms, future prospective applications and validation can help further develop the model.

As a common meteorological database, WorldClim is often used in ecological and GIS modeling. However, the disadvantage is that these data are a statistic of the past few decades, so there must be some errors in the recent data (58). For example, the current global warming, temperature and altitude are also changing, which will lead to additional effects. In order to solve the impact of these factors, we believe that real-time update of meteorological data will become very important in the future. In addition, some model features (such as slope, surface temperature and other terrain data) can also be added to improve the accuracy of model prediction.

## Conclusions

In this paper, PPR outbreak data combined with WorldClim database meteorological data were used to build a PPR prediction model. After the method of feature selection, eight sets of features were selected: bio3, bio10, bio15, bio18, prec7, prec8, prec12, and Alt for the modeling process. Then different machine learning algorithms were used to build models, among which the RF algorithm was found to have the best modeling effect. The ACC value of prediction accuracy of the model on the training set can reach 99.10%, while ACC on the test set is 99.10%. Therefore, the RF algorithm of eight variables was finally selected to build the model leading to the construction of the online prediction system. In addition, in order to determine the impact of each variable on the modeling results, we performed single-factor modeling and correlation analysis on the variables involved in the modeling. It was found that bio18 (the warmest quarterly precipitation), prec7 (the precipitation in July), and prec8 (the precipitation in August) contributed significantly to the model, and the outbreak of the epidemic may have an important relationship with precipitation. Eventually, we used the final qualitative prediction model to establish a global online prediction system for the PPR epidemic. On this basis, we hope that this research can play a role in monitoring the outbreak of PPR in the future. Thereby reducing the occurrence of epidemics and economic losses.

## Data Availability Statement

The raw data supporting the conclusions of this article will be made available by the authors, without undue reservation.

## Author Contributions

BN, QC, and XQ designed the study. RL, GZ, QZ, and QS collected the data. BN, RL, GZ, QZ, QS, XQ, and QC analyzed the data. All authors took part in interpreting the data and writing the manuscript.

## Conflict of Interest

The authors declare that the research was conducted in the absence of any commercial or financial relationships that could be construed as a potential conflict of interest.
