# Supplementary material for: Prediction for Global Peste des Petits Ruminants Outbreaks Based on a Combination of Random Forest Algorithms and Meteorological Data
Source: Front Vet Sci. 2021 Jan 7;7:570829. doi: 10.3389/fvets.2020.570829 (PMC7817769; doi:10.3389/fvets.2020.570829)
Supplement: Supplementary file 2 [file Data_Sheet_2.docx]

**Supplemental Data Sheet 2**

Prediction for global Peste des petits ruminants outbreaks based on a combination of random forest algorithms and meteorological data

Bing Niu^1#，*^，Ruirui Liang^1#^, Guangya Zhou^1^, Qiang Zhang^2^, Qiang Su^3^， Xiaosheng Qu^3*^,Qin Chen^1*^

**2.2 Methods**

**2.2.1 Naïve Bayes**

In this study, normal distribution was played.

**2.2.2 Random Forest**

The parameters are set as follows: the maximum depth of the trees is set to 0, the number of trees to be generated is set to 100 and the random number seed is to set 1.

**2.2.3 AdaBoost**

The parameters are set as follows: the number of iterations to be performed and is set to 10, the random number seed is set 1, the weight threshold for weight pruning is set to 100.

**2.2.4 Support Vector Machine**

The parameters are set as follows: the complexity parameter C is set to 1.0, PolyKernel is exponent =1. The number of folds for cross-validation used to generate training data for logistic models is set to -1, the random number seed is set to 1 and the tolerance parameter is 0.001.

**2.2.5 Artificial neural networks**

The parameters are set as follows: the learning rate is set to 0.3, Momentum applied to the weights is set to 0.2, the number of epochs to train through is set to 500, the Seed used to initialise the random number generator is set to 0, and the percentage size of the validation is set to 0.

**2.2.6 C4.5**

The parameters are set as follows: the confidence factor used for pruning is set to 0.25, the minimum number of instances per leaf is set to 2, the seed used for randomizing the data is set to 1.

**2.2.7 Feature selection**

In this study, we tried different combinations of attribute evaluator and search method to filter variables. Parameters are set to default.
